# In Spite of the Chemist’s Belief: Metastable Hydrates of CsCl

**DOI:** 10.1021/acsphyschemau.4c00093

**Published:** 2025-02-06

**Authors:** Kamila Závacká, Ľubica Vetráková, Johannes Bachler, Vilém Neděla, Thomas Loerting

**Affiliations:** † Institute of Scientific Instruments of the CAS, v.v.i., Kralovopolska 147, 61264 Brno, Czech Republic; ‡ Institute of Physical Chemistry, University of Innsbruck, A-6020 Innsbruck, Austria

**Keywords:** Cesium chloride hydrates, Freeze-concentrated solution, Cold-crystallization, Hyperquenching, Low-temperature X-ray diffraction, Environmental Scanning Electron Microscopy, Calorimetry

## Abstract

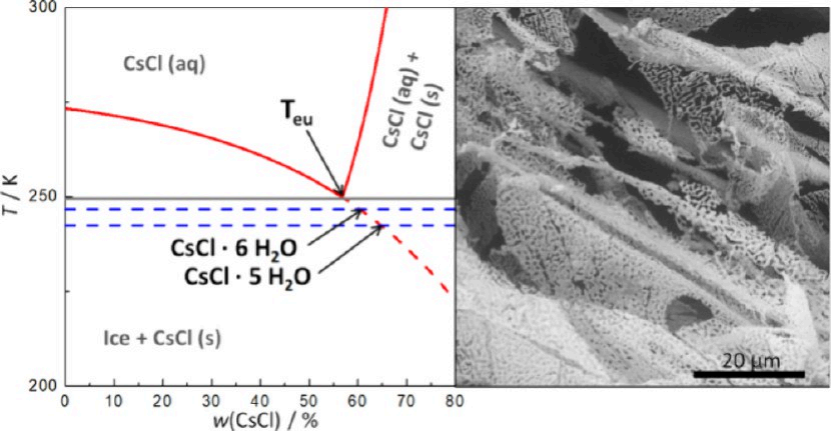

In this work, we focus on the low-temperature behavior of concentrated aqueous solutions of cesium chloride and discover two hydrates of CsCl. We employ four different methods, namely, (i) simple cooling at rates between 0.5 and 80 K s^–1^, (ii) simple cooling followed by pressurization, (iii) hyperquenching at 10^6^ to 10^7^ K s^–1^, and (iv) hyperquenching followed by pressurization. Depending on the method, different types of phase behaviors are observed, which encompass crystallization involving freeze-concentration, pressure-induced amorphization, full vitrification, and polyamorphic transformation. The CsCl hydrates discovered in our work cold-crystallize above 150 K upon heating after ultrafast vitrification (routes iii and iv) and show melting temperatures *below* the eutectic temperature of 251 K. We determine the composition of these hydrates to be CsCl·5H_2_O and CsCl·6H_2_O and find evidence for their existence in ESEM, calorimetry, and X-ray diffraction. The dominant and less metastable hydrate is the hexahydrate, where the pentahydrate appears as a minority species. We also reveal the birthplace for the CsCl hydrates, namely, the freeze-concentrated solution (FCS) formed upon cold-crystallization of the fully glassy solution (from iii and iv). The spongy FCS produced upon *cooling* of the liquid (from i and ii) is incapable of crystallizing CsCl-hydrates. By contrast, the FCS produced upon *heating* the glassy solution (from iii and iv) shows tiny, fine features that are capable of crystallizing CsCl-hydrates. Our findings contradict the current knowledge that alkali chlorides only have hydrates for the smaller cations Li^+^ and Na^+^, but not for the larger cations K^+^, Rb^+^, and Cs^+^ and pave the way for future determination of CsCl-hydrate crystal structures. The pathway to metastable crystalline materials outlined here might be more generally applicable and found in nature, e.g., in comets or on interstellar dust grains, when glassy aqueous solutions crystallize upon heating.

## Introduction

Currently, 10 alkali halide hydrates are known to form when freezing aqueous solutions.^1^ Probably the most common hydrate is hydrohalite (NaCl·2H_2_O). Besides NaCl, LiCl forms many hydrates at subzero temperatures including several metastable forms.^2,3^ The existence of hydrates is linked to the ionic radius of the alkali metal. Small cationic species tend to form hydrates, while larger ions do not.^4^ This explains why no hydrates of CsCl, KCl, and RbCl are known to form after freezing.^1,4−6^

For the CsCl-H_2_O system, the solid phase in equilibrium with the saturated solution is the anhydrous salt found in two polymorphic forms—the simple cubic form and the face-centered cubic form, which only plays a role at very high temperatures (above 745 K).^3^ Upon freezing aqueous CsCl solutions, first hexagonal ice forms. Ions are expelled since hexagonal ice is highly intolerant to impurities. The ions then end up in a freeze-concentrated solution (FCS). After reaching the eutectic temperature, which is reported to be from 250.7 K (−22.4 °C) to 248.3 K (−24.8 °C),^5−8^ the FCS crystallizes to CsCl and more ice crystals. A small portion of the FCS can also vitrify upon cooling, i.e., it turns into a noncrystalline, glassy solid rather than crystallize.^9^ This is typically incurred when the cooling rates are high, e.g., 100 K min^−1^^−1^ and above. Having a high atomic number, Cs is suitable for electron microscopic observation, providing excellent Z-contrast. The earlier ESEM observation of CsCl solutions at temperatures down to −27 °C shows the liquid FCS as well as crystallized CsCl after eutectic crystallization.^10^ The eutectic composition of CsCl+H_2_O is 7.83 M (56.87 wt %),^8^ 7.67 M (56.36 wt %),^5^ or 7.75 M,^6^ respectively.

The occurrence of hydrates can be inferred calorimetrically, especially upon heating the frozen solution. A frozen binary aqueous solution shows a broad single melting event if it is composed of ice and a freeze-concentrated solution only. Two melting events in the temperature range from 240 to 280 K typically take place if a hydrate is present in addition to ice. For instance, differential scanning calorimetry (DSC) scans for samples containing hexagonal ice and a NaCl hydrate show two melting events.^11^ In the case of CsCl, such calorimetry scans are lacking largely. DSC scans of hyperquenched CsCl at different molalities were reported by Hofer et al.^12^ Yet, they do not show the temperature range above 150 K, from which the presence of hydrates could be inferred.

The aim of the work is to study the vitrification and crystallization of aqueous CsCl solutions. In addition to the impact of the cooling rate on the process, we also study the impact of pressure in the range up to 1.6 GPa. This is because water features polyamorphism, where three different types of amorphous ice differing in terms of density exist in this range.^13^ Specifically, we use four different strategies: (i) simple cooling at ambient pressure, (ii) pressurization of simply cooled solutions,^14,15^ (iii) ultrafast cooling of micron-sized droplets in a vacuum chamber, referred to as hyperquenching,^16,17^ and (iv) pressurization of hyperquenched deposits.^18,19^ After pressurization (for ii and iv), the samples are quenched and recovered to ambient pressure while immersed in liquid nitrogen. This procedure retains the structure of the high-pressure sample at ambient pressure.^14^ The first strategy usually leads to crystallization. The third strategy produces a vitrified specimen of low-density (referred to as low-density amorphous ice, LDA), avoiding crystallization.^16,17^ The second and fourth strategies produce vitrified high-density samples (referred to as high-density amorphous ice, HDA). In the second strategy, HDA forms by pressure-induced amorphization of hexagonal ice, which forms first.^14^ In the fourth strategy, HDA forms from the polyamorphic transition from LDA.^18,19^ The states originating from routes ii, iii, and iv are highly metastable at ambient pressure so that possibly also metastable hydrates of CsCl might crystallize from them. According to Ostwald’s step rule,^20^ a highly metastable phase transforms to the stable phase in several steps via less metastable phases. This is because the less metastable phases form first owing to their lower activation barrier. We here investigate the question whether CsCl-hydrates are encountered in these steps using a combination of three methods, namely, electron microscopy, calorimetry, and X-ray diffraction.

## Methods

### Sample Preparation and Characterization

We chose a concentration of 0.5 M for the CsCl solution so that significant amounts of CsCl are present. The mother solution was prepared by dissolving CsCl in ultrapure water at room temperature. The following strategies were applied to prepare the frozen/vitrified solution:

(i) Samples cooled at ambient pressure for microscopic observation were prepared by pipetting a single droplet of 0.5 M CsCl solution ∼4–5 μm in diameter on an aluminum foil and by immersing it into liquid nitrogen. The foil was used to ensure a flat bottom of the sample, guaranteeing a better thermal contact with the cooling stage of the ESEM. For the DSC observation, a 16.11 mg droplet of 0.5 M CsCl solution was sealed into an aluminum DSC crucible and then cooled in the instrument as described later. For the XRD investigation, a few droplets of the solution were placed on a spoon, powdered, and then placed on the precooled sample holder (at 80 K). That is, the samples cooled in this way feature different cooling rates, depending on instrument. These range roughly from 30 K min^–1^ (for cooling inside the instrument) to about 500 K min^–1^ (for cooling in liquid nitrogen).

(ii) Pressure-amorphized samples were prepared using a piston–cylinder setup and a ZWICK BZ100/TL3S universal testing machine. 600 μL of the CsCl solution were pipetted into an indium container and placed inside the 8 mm diameter bore of the cylinder. The role of the indium container is to reduce friction between the sample and the piston–cylinder at cryogenic temperatures, as originally proposed by Mishima et al.^14^ The sample was then cooled to 77 K, compressed to 1.6 GPa, decompressed, and taken out of the piston–cylinder setup under liquid nitrogen. In this route, the sample crystallizes first at ambient pressure and is then amorphized above about 1 GPa at 77 K, known as pressure-induced amorphization. This represents the standard procedure that is used in the Loerting lab for about two decades to prepare amorphous samples of high density that allows recovery of the sample from the piston–cylinder setup, e.g., in ref (21).

(iii) For hyperquenched samples, we employed the setup of Kohl et al.,^17^ which results in cooling rates of approximately 1 million K s^–1^. Millions of droplets ∼3 μm in diameter made from CsCl solution were produced using an ultrasonic nebulizer and conveyed into a high-vacuum system through a 300-μm aperture with nitrogen as the carrier gas. The droplets were then deposited on an oxygen-free highly conductive copper substrate precooled to ≤80 K. After 30 min of deposition, a ∼3-mm-thick layer of a hyperquenched glassy solution was obtained. The vacuum was broken with dry nitrogen gas, and the substrate was quickly plunged into liquid nitrogen to recover it to ambient pressure.

(iv) For pressurized hyperquenched samples prepared from 0.5 M CsCl, the hyperquenched glassy sample described in iii was scratched off the copper substrate mechanically under liquid nitrogen, encapsulated in a cylindrical indium container, and transferred into the bore of the steel piston–cylinder setup. Using a ZWICK BZ100/TL3S universal testing machine, the sample was compressed to 1.6 GPa at 77 K, decompressed, and taken out of the piston–cylinder setup under liquid nitrogen. This follows the procedure that has recently been established in the Loerting lab in refs (18), (19), and (22).

For characterization of the samples using electron microscopy, calorimetry, and diffraction, small chunks were cut from the frozen sample and transferred under liquid nitrogen to the precooled instruments.

### Calorimetry (DSC)

To perform the calorimetric analysis, we used a PerkinElmer DSC 8000 differential scanning calorimeter. In the case of the sample cooled at ambient pressure (strategy i only), both heating and cooling scans were performed at a rate of 30 K min^–1^ inside the instrument. For the other cases (strategies ii, iii, and iv), the samples had to be loaded at cryo-temperatures to avoid conversion of the highly metastable samples. In the cold-loading procedure, such samples were loaded into the aluminum crucibles, and these were manually closed under liquid nitrogen and transferred to the precooled oven of the calorimeter. Each sample was heated to above 243 K (pressure-amorphized sample to 248 K, hyperquenched sample to 253 K, pressurized hyperquenched sample to 243 K) at 30 K min^–1^, then cooled to 100 K, and again heated to 313 at 30 K min^–1^. Knowing the mass of the samples is necessary to normalize the calorigrams. However, weighing of the cold-loaded samples is not possible due to the requirement to keep them under liquid nitrogen. Therefore, the heat of fusion for a 0.5 M CsCl solution (211 J g^–1^) was determined in the simple cooling experiment from the area of the ice melting peak and the mass of the loaded liquid sample. The masses of the cold-loaded samples were determined from the area under the ice melting endotherm of each individual sample divided by the heat of fusion, and the traces were normalized to the unit mass. The ordinates are shown in units of W g^–1^, with the exothermic events oriented downward.

### Environmental Scanning Electron Microscopy (ESEM)

The microscopic images were recorded by a noncommercial ESEM AQUASEM II redesigned from a Tescan SEM VEGA.^23^ For low temperature measurements, the ESEM is equipped with a specially designed cryostage that can reach temperatures down to 80 K, or a Peltier-cooled sample holder, reaching temperatures down to 223 K.

The samples were quickly transferred from LN_2_ onto the precooled stage of the ESEM to avoid their heating and frost formation on the surface. The observation was conducted in the pressure range 100–300 Pa of the mixture of nitrogen and water vapor. The electron beam energy of 20 keV and currents of 64–125 pA were applied to scan the sample, with the backscattered electrons detected by a YAG:Ce^3+^ scintillation detector,^24^ sensitive to the material composition of the sample.

### Low-Temperature X-ray Diffraction (XRD)

Diffractograms were recorded on a D8 Bruker Advance with a Cu Kα radiation source (λ = 0.154178 nm) and a Goebel mirror. The diffraction patterns were resolved by using a high-resolution LynxEye XE-T array detector. Temperature control between 20 and 300 K was achieved using a cryochamber manufactured by FMB Oxford Ltd., which employs a two-stage He cryopump and resistive heating elements. All samples were analyzed using copper sample holders, so the peaks of Cu are visible in the XRD diffractograms. The hyperquenched sample had been deposited directly on a specially designed copper sample holder in order to easily transfer the sample to the precooled instrument (80 K). The pressurized samples were powdered under liquid nitrogen and placed onto the precooled sample holder inside the instrument at 80 K. Similarly, the simply cooled samples were placed on a spoon, immersed in liquid nitrogen, powdered on the spoon, and transferred onto the precooled sample holder.

## Results

We have investigated the structure and properties of the frozen samples by a combination of three characterization techniques: differential scanning calimetry (DSC), low-temperature X-ray diffraction (XRD), and environmental scanning electron microscopy (ESEM).

### Differential Scanning Calorimetry (DSC)

**Figure 1:**
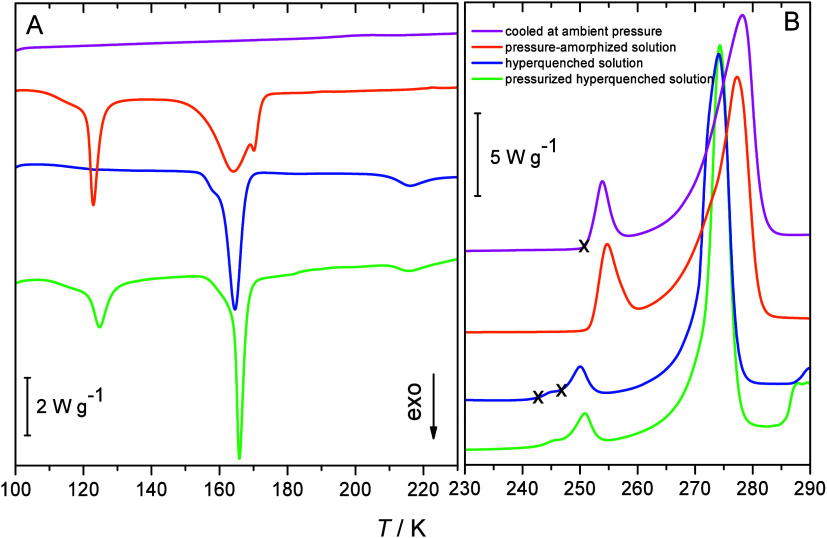
Calorimetry scans of frozen 0.5 mol/L CsCl aqueous solution recorded at a 30 K min^–1^ heating rate, (A) at 100–225 K, featuring the polyamorphic HDA/LDA transition near 120 K, cold crystallization of the amorphous sample near 160 K, and the polytypic transition from ice *I*_sd_ to I_h_ near 210 K, and (B) at 230–290 K, featuring the melting endotherms of CsCl/ice eutectic at 250 K (marked by X) and melting endotherms for metastable CsCl-hydrates (marked by two x) as well as ice melting immersed in CsCl solution (above 260 K). The temperatures labeled “X” are 242.5, 246.7, and 250.7 K.

Figure 1 shows DSC heating traces of frozen 0.5 M CsCl samples prepared by the four above-mentioned freezing procedures (cooled at ambient pressure, pressure-amorphized, hyperquenched, and pressurized hyperquenched). Figure 1A reveals one major difference between the pressurized and unpressurized samples: the exotherm near 120 K. This exotherm is indicative of the sample being in a high-density amorphous state (HDA) that transforms to a low-density amorphous state (LDA). This is followed by the crystallization from LDA to stacking-disordered ice (*I*_sd_) starting at ∼150 K. Also, the hyperquenched sample shows the crystallization exotherm, meaning that the hyperquenching technique was successful in avoiding crystallization and achieving full vitrification of the CsCl solution. By contrast, the simply cooled solution merely shows a baseline below 240 K, meaning that cooling at 30 K min^–1^ leads to crystallization but not vitrification. Small exothermic peaks can be seen starting at ∼210 K in the case of hyperquenched and pressurized hyperquenched samples, which indicate the polytypic transition from ice *I*_sd_ to stable hexagonal ice (ice I_h_).^25^ That is, after hyperquenching, ice *I*_sd_ initially crystallizes from the vitrified solution. After pressure-induced amorphization of the frozen solutions (second trace from top in Figure 1A), much less stacking disorder and cubicity develops as indicated by the very weak and broad exotherm (barely seen in Figure 1A). By, contrast the simple cooling approach leads to hexagonal ice, where stacking disorder and cubicity are absent entirely (no exotherm at all in the top trace in Figure 1A). While Figure 1A (temperature range 100–225 K) features baseline and exotherms only, Figure 1B (temperature range 230–290 K) features endotherms that are assigned to melting events of crystalline parts. All thermograms showcase a massive endotherm near 270 K that displays fronting. The onset of the fronting is at ∼260 K, possibly even below but superposed with another endotherm. Such fronting is typical of the melting event of hexagonal ice that is immersed in a freeze-concentrated salty solution. The salty solution decreases the ice melting point. As more and more ice melts, the salty solution gets more and more diluted, so that the melting point shifts continuously to higher temperature. This continues as long as all ice has melted, at which point the equilibrium melting point of the salt solution of the original concentration (without freeze concentration) has been reached.

Superposed with the fronting of the ice I_h_ melting endotherm, we observe weaker endotherms. These endotherms appear at three different onset temperatures, each marked by an X in Figure 1B. Depending on the sample preparation method, either one additional or two additional endotherms are seen. For the simply cooled, nonvitrified solution (top trace in Figure 1B) and the pressure amorphized solution (second trace from top), the onset temperature of the first endothermic peak is ∼250 K. This temperature corresponds to the eutectic temperature of CsCl/ice (250.7 K, Gao et al.^6^). For hyperquenched (2^nd^ trace from bottom) and pressurized hyperquenched samples (bottom trace), a double peak occurs with onsets at ∼242 and ∼247 K. These represent two melting events for noneutectic crystals.

The mere observation that the melting temperature is *below* the eutectic melting temperature implies that the phases that melt are not equilibrium phases—equilibrium phases in an eutectic phase diagram always melt at temperatures *above or at* the eutectic line. Consequently, the phases melting below 250 K are metastable phases. For other salts, such as NaCl or LiCl, this is the typical temperature range, in which different types of hydrates such as NaCl·2H_2_O or LiCl·H_2_O melt. We, therefore, tentatively assign the two melting events for the hyperquenched and pressurized hyperquenched samples to two CsCl-hydrates, which are metastable with respect to the eutectic ice/CsCl mixture. This hydrate would then melt at the extrapolated liquidus line in the binary phase diagram and hence be richer in CsCl and poorer in H_2_O compared with the eutectic composition. In other words, the hydrate exceeds the solubility limit due to the hyperquenching procedure. The eutectic is located at a molality of 7.8 mol/kg of CsCl, which corresponds to the solubility limit at 250.7 K.^6^ At room temperature the solubility of CsCl in water is about 11 mol/kg. Cold-crystallization of the hyperquenched, vitrified 0.5 M CsCl solution upon heating the glass would then lead to a freeze-concentrated solution of a CsCl molality >7.8 mol/kg. Since 1 kg of water contains 55 mol of H_2_O, the CsCl:H_2_O ratio is 1:<7.1, meaning that CsCl·7H_2_O, CsCl·6H_2_O, and CsCl·5H_2_O are possible candidates for these metastable hydrates. In order to test this hypothesis and tentative assignment, we have carried out X-ray diffraction and electron microscopy experiments as detailed below.

### X-ray Diffraction

**Figure 2:**
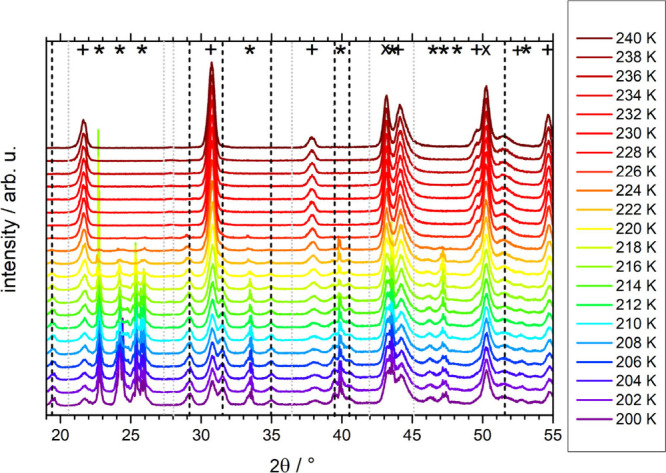
Powder X-ray diffractograms for 0.5 M CsCl cooled at ambient pressure by plunging into liquid nitrogen. Samples were cold-loaded at 80 K and then slowly heated at about 1 mbar to 240 K. Each diffractogram takes about 20 min to be recorded. The plus symbols (+) indicate Bragg peaks for bare CsCl; the asterisks (*) indicate ice Bragg peaks for a temperature of 220 K, and X indicates the peak of the copper holder. Vertical dashed lines indicate Bragg peaks for the unknown phase, which we attribute to be a volatile hydrate of CsCl: the positions of the peaks that are present in this sample are colored black, the positions of the hydrate peaks from other samples (Figures 3–5) are colored gray.

**Figure 3:**
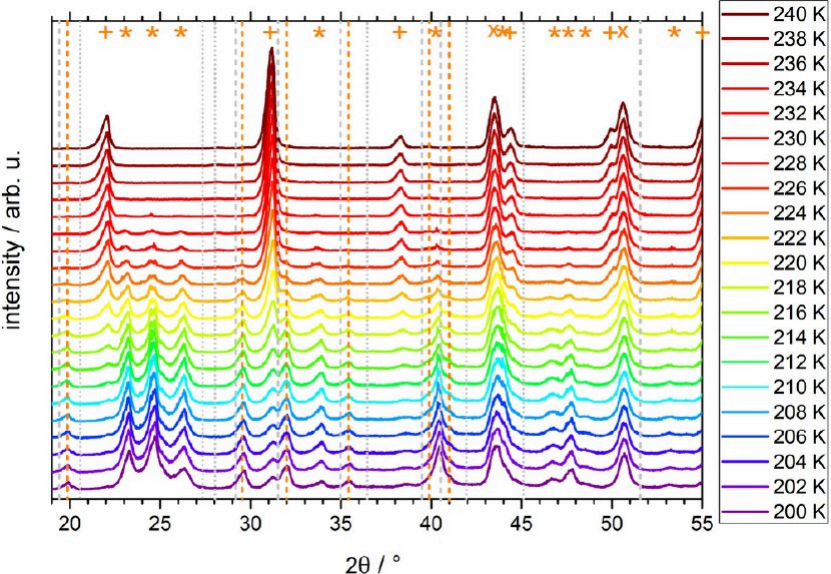
Powder X-ray diffractograms (same as Figure 2) for a pressure-amorphized 0.5 M CsCl solution. About 3 mbar of atmosphere remain in the chamber in this experiment. All the peaks are shifted by 0.4 to the right in comparison with the other samples. Vertical dashed lines indicate Bragg peaks for the unknown phase, which we attribute to being a volatile hydrate of CsCl: the positions of the peaks that are present in this sample are colored orange due to the shift of all the peaks by 0.4 to the right; the positions of the peaks of the other samples are colored gray.

**Figure 4:**
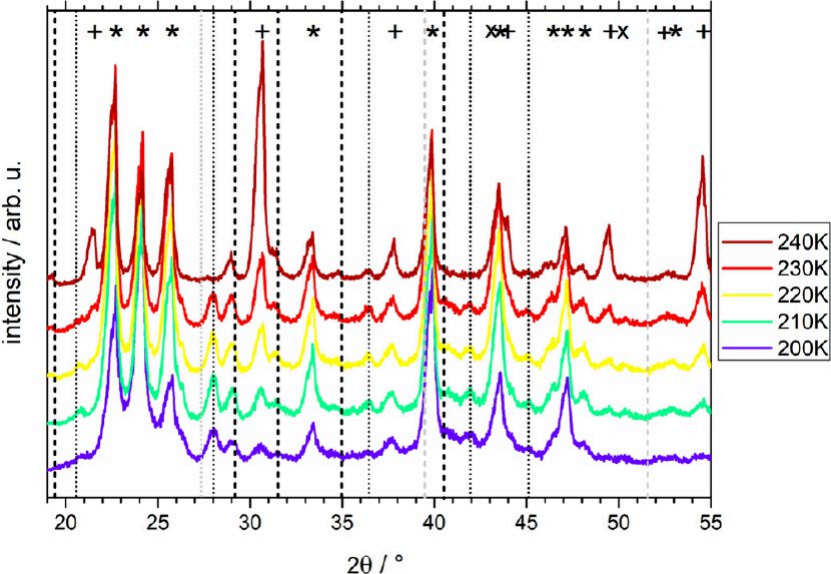
Powder X-ray diffractograms (same as Figure 2) for a hyperquenched 0.5 M CsCl solution. About 10 mbar of dry air were put, on purpose, into the chamber to avoid ice sublimation. Vertical lines indicate Bragg peaks for the unknown phase, which we attribute to being a volatile hydrate of CsCl: the positions of the peaks that are present in this sample are colored black; the positions of the peaks of the other hydrate are colored gray. Dashed lines mark the peaks of the “first” hydrate, dotted lines mark the peaks of the “second” hydrate.

**Figure 5:**
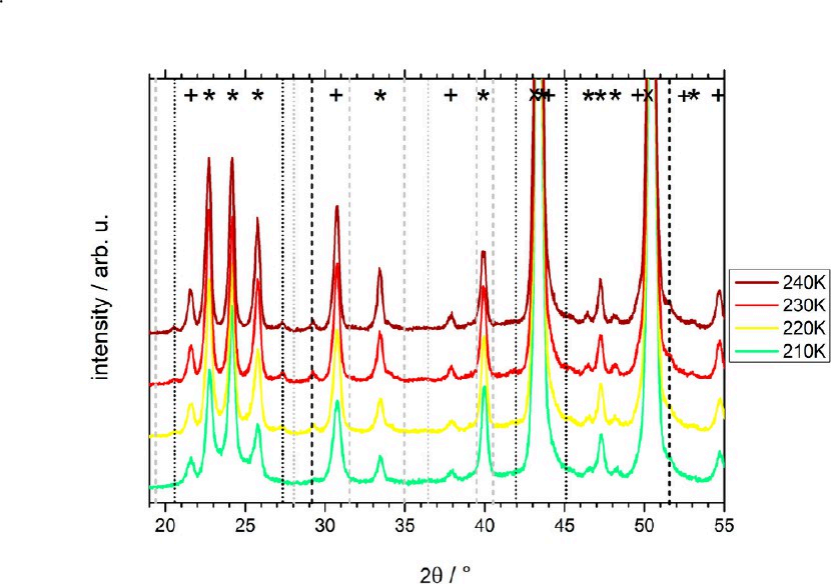
Powder X-ray diffractograms (same as Figure 2) for a pressurized hyperquenched 0.5 M CsCl solution. About 10 mbar of dry air were put, on purpose, into the chamber to avoid ice sublimation. The massive Bragg peaks marked by the symbol X arise from the Cu-sample holder that is not fully covered with sample. Vertical lines indicate Bragg peaks for the unknown phase, which we attribute to being a volatile hydrate of CsCl: the positions of the peaks that are present in this sample are colored black; the positions of the peaks of the other samples are colored gray. Dashed lines mark the peaks of the “first” hydrate; dotted lines mark the peaks of the “second” hydrate.

The hypothesis for a metastable hydrate of composition CsCl·≤7H_2_O is tested based on X-ray diffraction measurement of frozen 0.5 M CsCl solutions prepared by all mentioned methods. Powder X-ray data obtained upon heating (just like in the calorimetry scans in Figure 1) are shown in Figures 2–5 in the temperature range 200–240 K, just before the melting temperature of the presumed metastable hydrates from the calorimetry experiment. The X-ray data show Bragg peaks arising from two known phases belonging to the sample: hexagonal ice (marked by *) and CsCl (marked by +), and from the copper sample holder (marked by X). The positions of the hexagonal ice peaks were taken from the literature^26,27^ and recalculated to 220 K (the median of our temperature range) using the lattice constants for ice I_h_ at 220 K.^27^ The ice peaks are located at 22.7°, 24.2°, 25.8°, 33.5°, 39.9°, 43.6°, 46.4°, 47.2°, 48.2°, and 53.1°. The positions of bare CsCl peaks at 220 K were calculated using *hkl* indexes^28^ and a temperature-dependent lattice parameter.^29^

The sample cooled simply at ambient pressure was placed on a spoon, plunged into liquid nitrogen, powdered, and then cold-loaded to the sample holder, which was then evacuated to about 1 mbar. That is, the cooling rates are somewhat higher here than in the calorimetry experiment reported in Figure 1. Yet, the X-ray diffractograms in Figure 2 clearly show that the sample has not vitrified but crystallized as well at the higher cooling rate (of roughly 500 K min^–1^ instead of 30 K min^–1^). Above 216 K, the hexagonal ice Bragg peaks start to shrink without any halo peak typical of liquid water appearing, indicating sublimation of ice in the vacuum chamber. Up to 228 K, remnants of hexagonal ice can be identified. At 230 K and above, CsCl is the only phase that remains on the sample holder.

Subtracting the known phases from the diffractogram at 200 K shows that several weak Bragg peaks remain. These are located at 19.4°, 29.2°, 31.5°, 35.0°, 39.5°, 40.5°, and 51.6°, and marked by black vertical dashed lines in Figure 2. Similar to hexagonal ice, they disappear above 230 K by sublimation (except for the one at 51.6°), which indicates that the new unidentified phase to which these Bragg peaks belong is a volatile phase. Furthermore, the vapor pressure of this volatile phase is quite similar to the vapor pressure of hexagonal ice. That fits very nicely with what one would expect from a salt hydrate. That is, plunging into nitrogen and cooling rates of about 500 K min^–1^ produce a small amount of metastable hydrate of CsCl, as opposed to the observation in the calorimeter after cooling at 30 K min^–1^. The calorigram merely features CsCl/ice eutectic melting, i.e., the phases marked by + and * in Figure 2.

The phenomenology is similar for the pressure-amorphized sample. First, we need to notice that all the peaks in the calorigram of the pressure-amorphized sample, whether they are assigned to the ice, CsCl, salt hydrate, or copper holder, are shifted by approximately 0.4° toward higher values compared to the peak positions in the diffractograms of samples prepared in other ways. For that reason, we have also shifted the marking symbols (+, *, X and vertical dashed lines) by 0.4° to the right to fit the corresponding peaks and colored them differently (orange). At 200 K the Bragg peaks for CsCl are absent (e.g., at 22.1°, 38.4°, 44.4°, and 50.0°) or diminished (at 31.1°). Yet, the additional Bragg peaks similar to the ones in Figure 2 are present also in Figure 3 at 200 K (orange vertical lines). That is, the Bragg peaks that we assign to salt hydrate are present but the bare salt is not. The peaks belonging to CsCl salt start to appear above 210 K. At higher temperatures, hexagonal ice and hydrate water sublime, leaving CsCl behind. A slight difference is that the ice I_h_ peaks remain up to 232 K, which is due to a slightly weaker vacuum (ca. 3 mbar) that results in higher sublimation temperature. That is, in this case, the pressurization procedure avoids bare CsCl, as opposed to the simple cooling approach without pressurization.

Let us now inspect X-ray data of hyperquenched and pressurized-hyperquenched solutions in Figures 4 and 5, respectively. In Figure 4, there are extra Bragg peaks at 20.6°, 28.0°, 29.2°, 31.5°, 36.4°, 40.5°, 41.9°, and 45.1° at 200 K, i.e., after cold-crystallization of the hyperquenched sample (the extra peaks at 19.4° and 35.0° appear at higher temperatures—above 230 K). This again speaks for the presence of the crystalline CsCl-hydrate. Yet, some of these Bragg peaks are located at positions similar to those noted in Figures 2 and 3 (marked by black dashed lines), but others appear at new positions, e.g., 28.1°, 36.4°, and 41.9° (marked by black dotted lines). This suggests the presence of at least two different hydrates. Some of the “new” hydrate Bragg peaks (seen in Figure 4) disappear above 240 K, while the others (36.4° and 45.1°) and the “old” hydrate Bragg peaks (known from Figure 3) do not disappear. Bare CsCl Bragg peaks are seen even at the lowest temperature of 200 K, and they enlarge as the temperature rises. We attribute the disappearing Bragg peaks to a second metastable hydrate(s) CsCl·≤7H_2_O. The first metastable hydrate remains in this experiment, which was done in a 10 mbar vacuum (i.e., above the triple point of ice, so that ice sublimation is prevented). The fact that hexagonal ice and the first hydrate remain, while the second one sublimes at 240 K indicates their vapor pressures below 10 mbar for the former, but above 10 mbar for the latter. The second metastable hydrate is of higher vapor pressure and sublimes first; it is the most metastable hydrate. This corresponds to the hydrate melting at ∼242.5 K in the calorimeter in Figure 1. The first metastable hydrate corresponds to the one melting at ∼246.7 K in the calorigram in Figure 1. That is, just like in the calorigrams, we also see evidence for two metastable hydrates in the diffractograms. The low intensities of the Bragg peaks for the second, most metastable hydrate in the diffractograms agree nicely with the low area of the melting peak in the calorigram in Figure 1. That is, only a very small amount of the second hydrate forms but a larger amount of the less metastable first hydrate.

In Figure 5, the phenomenology is similar for the pressurized hyperquenched sample. Again, Bragg peaks attributed to CsCl-hydrates appear, e.g., at 27.3°, 29.2°, 45.1°, and 51.6°, and also bare CsCl is seen starting from 210 K. In this experiment, again conducted in about a 10 mbar atmosphere, the hydrate persists at least up to 240 K, just like hexagonal ice does.

**Figure 6:**
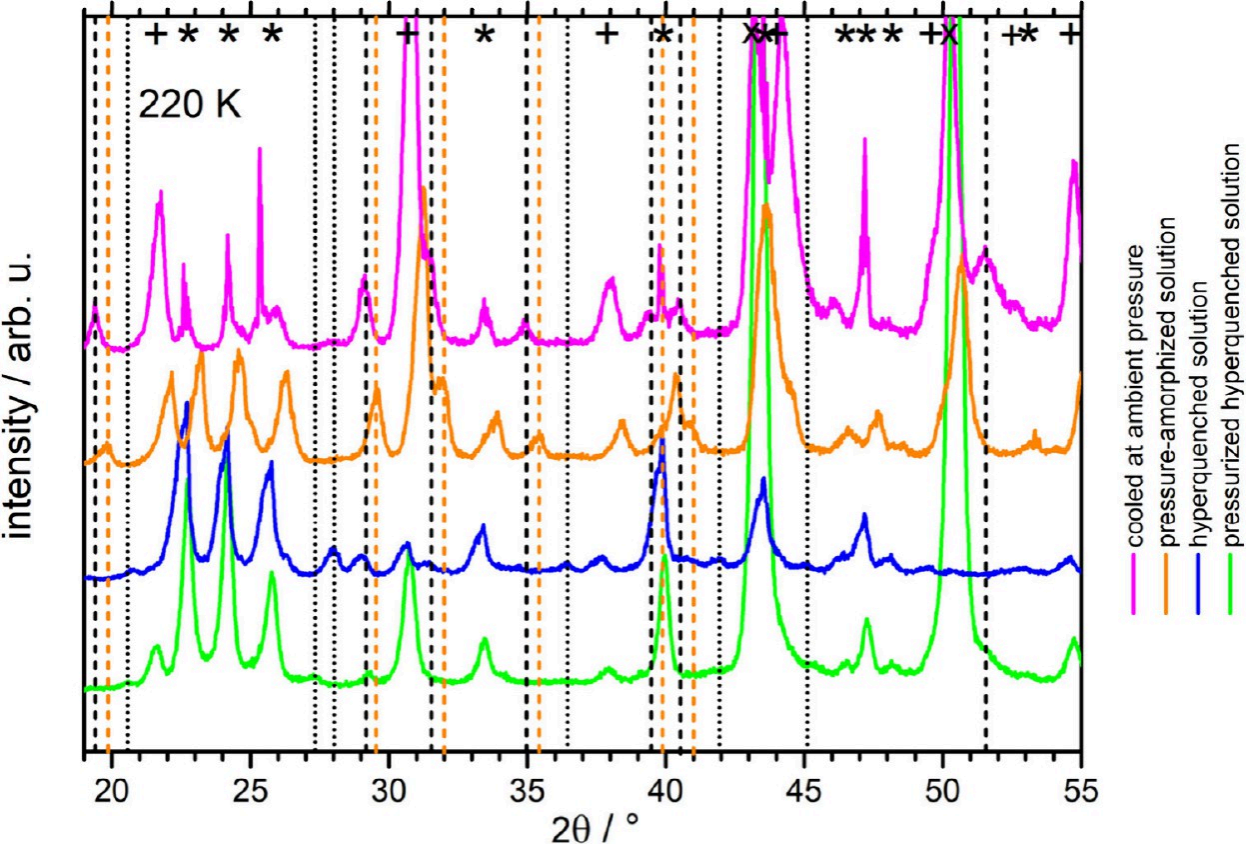
Comparison of the four distinct preparation routes at 220 K. The X-ray diffractograms of 0.5 M CsCl cooled at ambient pressure by immersing into liquid nitrogen (magenta), pressure amorphized (orange), hyperquenched (blue), and pressurized hyperquenched (green) solution. Bragg peaks for the hexagonal ice are labeled “*”; for CsCl crystals, “+”; for the Cu sample holder, “×”. Vertical lines indicate Bragg peaks for the unknown phase, which we attribute to being a volatile hydrate of CsCl: the positions of the peaks in pressure amorphized-solution are colored differently (orange) due to them being shifted by 0.4° to the right relative to the peaks of other samples. Dashed lines mark the peaks of the “first” hydrate; dotted lines mark the peaks of the “second” hydrate.

While the observations of the novel Bragg peaks are clear, the number of identified Bragg peaks is quite low for a crystal structure refinement. So, at this moment, we can only conclude qualitatively from the X-ray results that (at least) two volatile hydrates are present for the hyperquenched samples, but only one seems to be present for the sample cooled simply in liquid nitrogen. Bragg peaks related to these hydrates are marked with dashed and dotted vertical lines in Figure 6, which summarizes the XRD scans of all types of samples at 220 K as an overview. We detect the new form at 200–226 K in the case of a sample cooled at ambient pressure and a pressure amorphized sample (at higher temperatures, the water from the hydrate already sublimated due to low pressure conditions), at 200–240 K in the case of hyperquenched sample, and at 220–240 K in the case of pressurized hyperquenched solution. That is, a good observation range for the metastable hydrates is 200–240 K, and so we have used this temperature range for our microscopy study.

### Environmental Scanning Electron Microscopy (ESEM)

**Figure 7:**
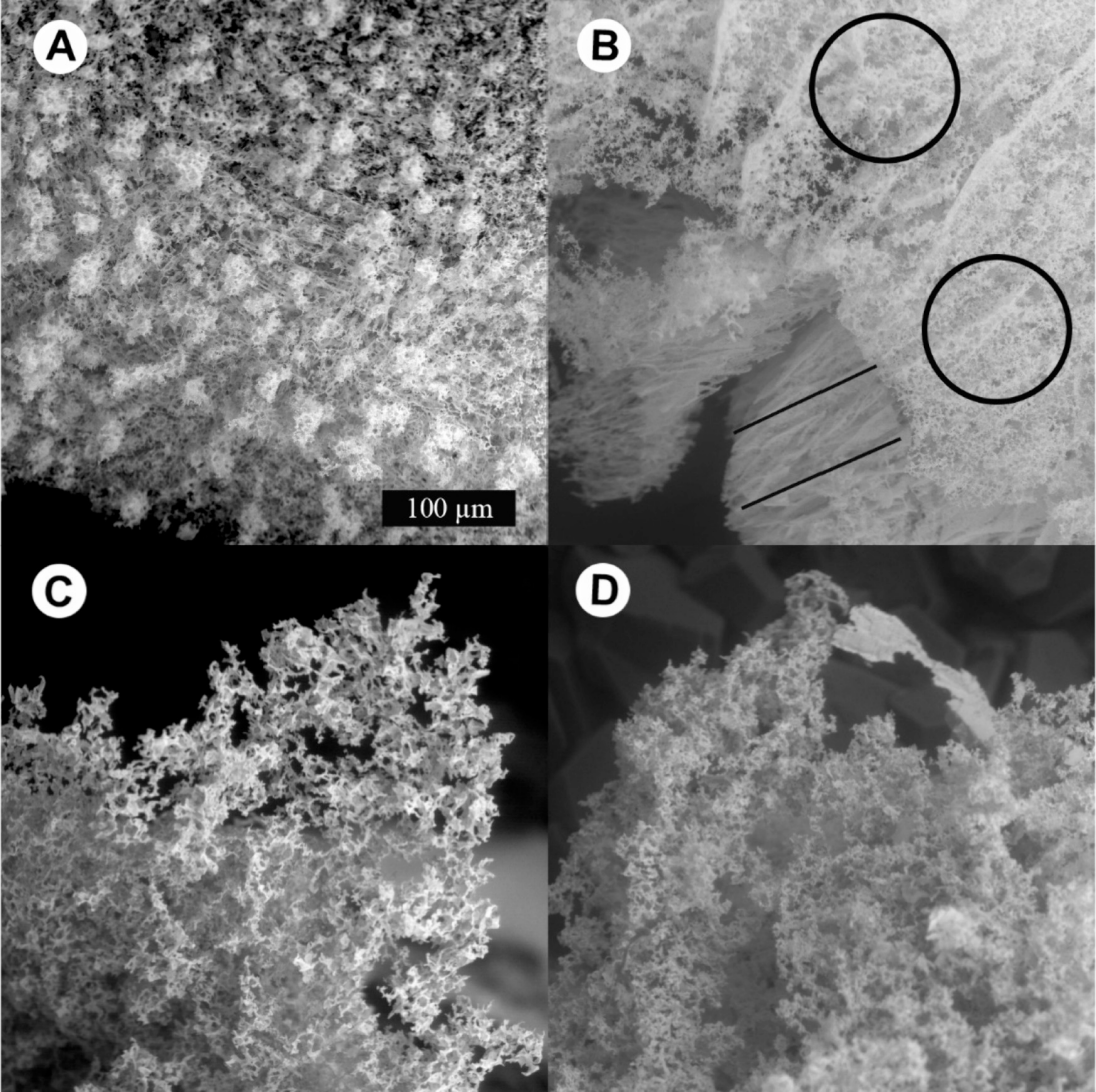
ESEM micrographs of frozen 0.5 M CsCl aqueous solution recorded by the BSE detector. The measurement conditions were as follows: for “A, solution cooled at ambient pressure,” the temperature of the cooling stage was set to 223 K, N_2_ pressure to 300 Pa, and H_2_O pressure to 0 Pa; for “B, pressure-amorphized solution,” the temperature of the cooling stage was set to 233 K, N_2_ pressure to 300 Pa, and H_2_O pressure to 13 Pa; for “C, hyperquenched solution,” the temperature of the cooling stage was set to 223 K, N_2_ pressure to 310 Pa, and H_2_O pressure to ∼5 Pa; and for “D, pressurized hyperquenched solution,” the temperature of the cooling stage was set to 233 K, N_2_ pressure to 300 Pa, and H_2_O pressure to 15 Pa.

**Figure 8:**
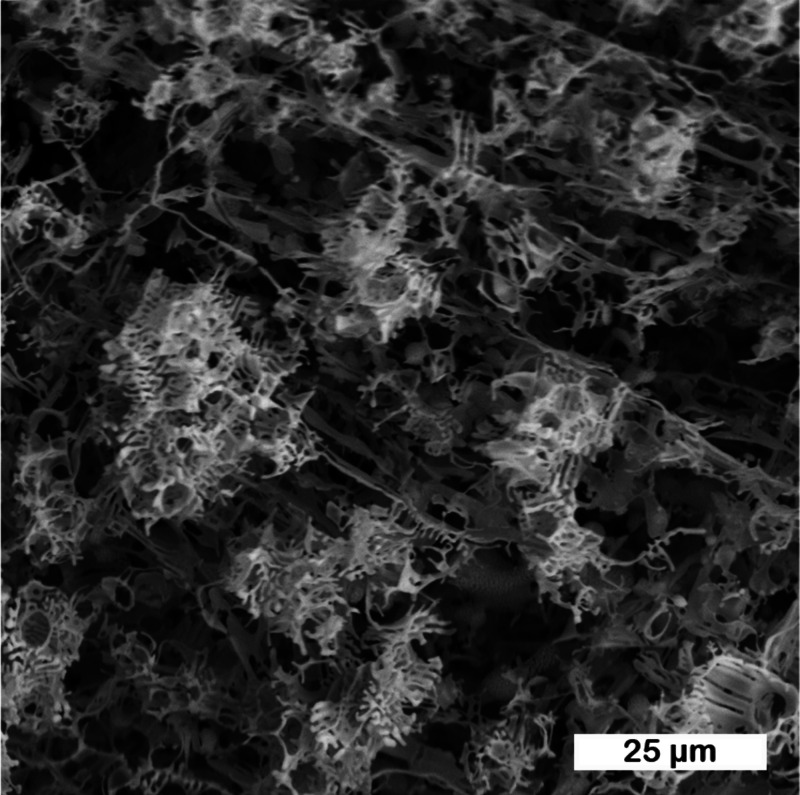
Magnification of Figure 7A: 0.5 M CsCl solution cooled at ambient pressure at 77 K, observed at 223 K after hexagonal ice sublimation.

**Figure 9:**
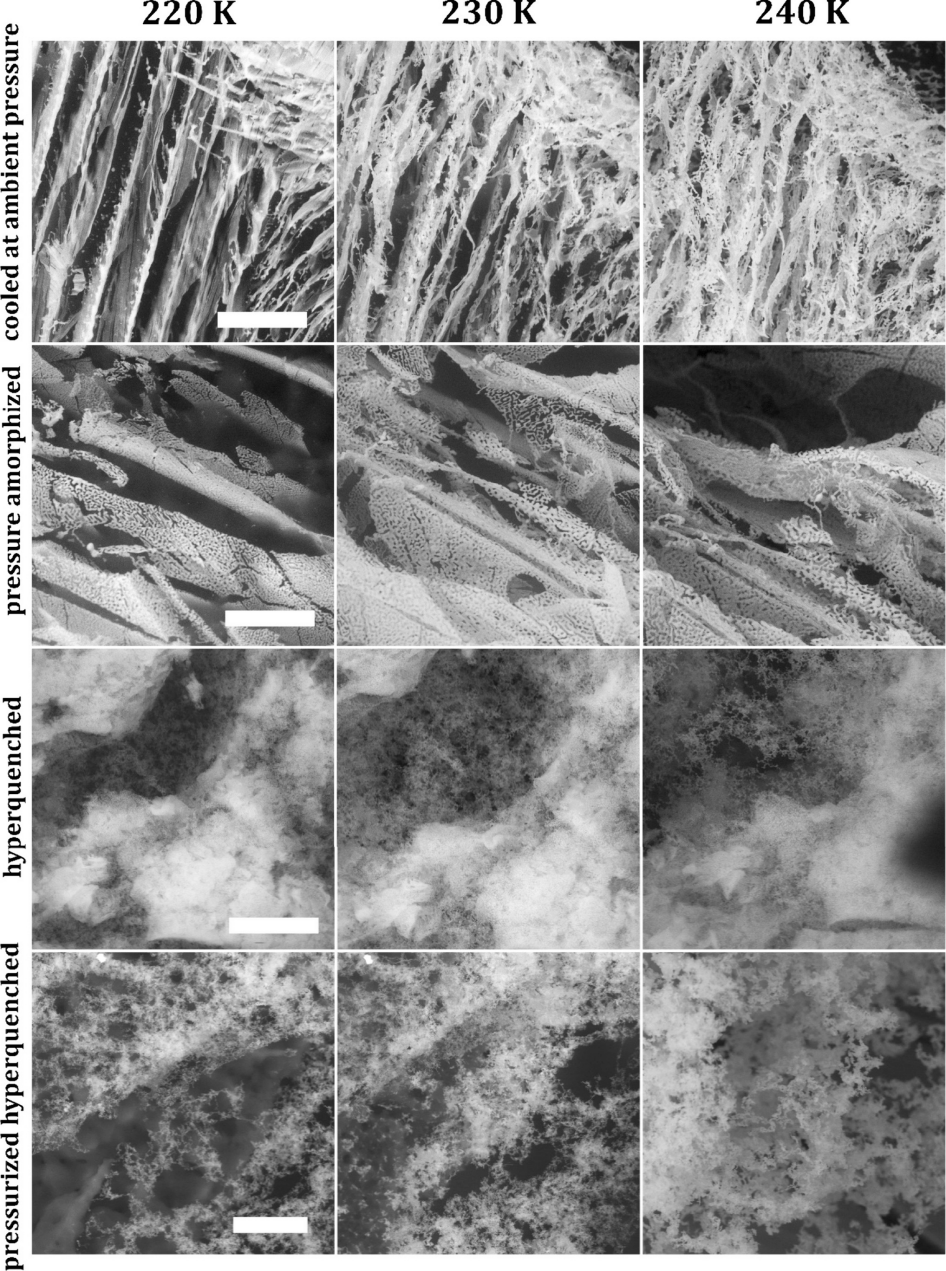
ESEM micrographs of 0.5 M CsCl solution prepared by all four methods recorded at temperatures 220, 230, and 240 K with revealed CsCl structure after ice sublimation. The scale bar represents 20 μm.

The structures of frozen CsCl samples prepared by the four presented methods were studied in the ESEM. Figure 7 shows ESEM micrographs of 0.5 M CsCl aqueous solution frozen in four different ways quite similar to the ones mentioned above (A, solution cooled at ambient pressure in the liquid nitrogen; B, pressure-amorphized solution; C, hyperquenched solution; and D, pressurized hyperquenched solution). Cooling at ambient pressure was done by immersing in liquid nitrogen rather than slow cooling inside the instrument, similar to the X-ray diffraction case. Micrographs were recorded at 223 K (Figure 7A/C) or 233 K (Figure 7B/D), similar to the X-ray diffractogams in Figure 6, and in the range in which X-ray diffraction and calorimetry suggest the existence of CsCl hydrates. Figure 8 shows a magnification of Figure 7A. Figure 9 depicts the temperature-induced morphology changes of 0.5 M CsCl samples prepared by the four discussed methods. The samples were loaded on the cryostage precooled to approximately 80 K, the temperature was raised gradually, and the structural changes were monitored in the temperature range of 80–260 K. Let us now look to the morphology in these microscopy images that show a comparison for all four pathways at 220, 230, and 240 K. The micrographs show intensities of detected backscattered electrons’ (BSE) signal, which are directly proportional to the atomic number of an element in the sample. This results in a material contrast (Z-contrast) that allows us to localize the position of the salt containing Cs (the bright areas). Ice and hydrates have already sublimated from the observed samples due low water vapor pressure in the specimen chamber.

In the micrographs, we see the structure of the salt crystals and the relief of the sample. In all samples, we see the “spongy” structure of the CsCl crystals, which remains on the cooling pad after ice and hydrate sublimation. This is seen in magnification in Figure 8 more clearly. In the case of the sample cooled in liquid nitrogen and then pressurized (Figure 7B), we distinguish two types of crystal structures: a sparse “sponge-like” structure marked by black circles and a denser crystal structure of the long solid grain boundaries marked by black lines. The former features the same CsCl-structure after evaporation of hexagonal ice known from Figure 7A. The latter could be the relief of the FCS before sublimation of “secondary ice”^30^ (i.e., ice in the solidified FCS). The observed FCS most probably involves CsCl-hydrate that has formed previously due to devitrification of the pressure-amorphized solution, as observed in the X-ray measurements (Figure 3). Before observation of sample D, it changed its volume and exploded, which could influence its inner crystalline structure.

In Figure 9, we can see the change of the CsCl FCS crystalline structure upon temperature change from 220 to 240 K. We can see that the salt structures are thicker and arranged in parallel planes in the sample cooled at ambient pressure and in the pressure-amorphized sample. During these cooling procedures labeled as i and ii, the water crystallizes to I_h_ forming relatively big parallel ice grains.^9,30^ The ice grains separate FCS (concentrated CsCl solution) at its grain boundaries. Therefore, the linear features seen in the first two samples in Figure 9 are given by the morphology of the already sublimed “primary ice.”^30^

On the other hand, the salt in hyperquenched and pressurized-hyperquenched samples forms a fluffy texture similar to cotton wool, and it was difficult to focus on it. This is again given by the morphology of the already sublimed ice from the sample. During the ultrafast cooling procedure labeled as iii, small droplets of CsCl solution vitrify, leading to a less organized CsCl location in the sample. Upon heating of the vitrified droplets, the small I_h_ crystals are formed due to the cold-crystallization event. This event expels CsCl ions from the numerous tiny I_h_ crystals and produces the freeze-concentrated solution upon warming (as opposed to the freeze-concentrated solution formed upon cooling for the simple cooling experiment). After sublimation of ice, the structure of the FCS is uncovered having the “fluffy” texture.

The most apparent structural change is detected in the sample cooled at ambient pressure, where the parallel planes transform into “sponge-like” structure upon heating. That is, we see the sublimation of “secondary” hexagonal ice^30^ (i.e., ice in the solidified FCS) in this temperature range. The fluffy nature is unique for both hyperquenched types of samples. Also, the calorigrams in Figure 1 are unique for those two samples, showcasing the metastable hydrates.

## Discussion and Conclusions

We here reveal evidence for the existence of metastable hydrates of CsCl, in spite of the belief that hydrates do not exist for such large cations. While hydrates of LiCl and NaCl are known, so far none are known for KCl, RbCl, and CsCl. A hydrate of Cs^+^, being the largest cation in the alkali metal group, thus comes as a surprise. Despite the fact that the existence of CsCl hydrate was previously contradicted in the literature due to the absence of characteristic peaks in terahertz spectra,^1^ our collected evidence demonstrates the existence of crystalline hydrates of CsCl. Both hydrates are obtained particularly for solutions that are first turned into a fully glassy state by hyperquenching and then cold-crystallized upon warming. Solutions that crystallize upon simple cooling, on the other hand, produce a eutectic ice/CsCl mixture, with some hydrates after plunging into liquid nitrogen and no hydrates at all after slow cooling, instead. That is, the freeze-concentrated solution that cold-crystallizes upon heating differs inherently from the freeze-concentrated solution obtained upon cooling. The nature of the cold-crystallized FCS is much fluffier in the ESEM images, suggesting that the tiny, fine nature of the veins and network of cold-crystallized FCS might be at the origin of the CsCl-hydrate formation.

The first piece of evidence for the existence of the CsCl-hydrates is the appearance of subeutectic melting endotherms in calorigrams (Figure 1) for hyperquenched samples, i.e., samples that were initially vitrified at cooling rates of 10^6^ to 10^7^ K s^–1^ and subsequently crystallized by heating. The very high chemical potential of a fully vitrified solution represents the key to this discovery. From this starting point, the transition to the thermodynamically stable eutectic CsCl plus ice upon heating takes place in steps, where we identify two distinct melting temperatures, i.e., two distinct metastable hydrates. That is, the kinetic barrier to produce these hydrates is low, and so two metastable phases of higher chemical potential than the eutectic mixture form first, in full accordance with the suggestions made by Ostwald when phrasing his step rule.

The second piece of evidence for the presence of these hydrates is the observation of unknown Bragg peaks in the X-ray diffractograms. These Bragg peaks appear side-by-side with hexagonal ice and bare CsCl at 200 K. Just like hexagonal ice, the hydrate Bragg peaks disappear due to sublimation (if the total pressure in the chamber is about 1 mbar) or due to conversion to the eutectic mixture (if the total pressure is 10 mbar). The sublimation of hydrates slightly before the sublimation of ice is again indicative of the volatile nature typical of a hydrate (as opposed to nonvolatile salt), and the higher vapor pressure is indicative of its higher chemical potential—recalling Ostwald’s idea. The rather low number of Bragg peaks for the hydrates and the simultaneous observation of ice and CsCl do not allow us to extract the crystal structure and hydrate composition from the X-ray data.

The third piece of evidence is provided by ESEM micrographs, in which we see the relief of phases of different morphologies that have evaporated in the remaining CsCl. Specifically, sublimation of the metastable hydrates cold-crystallized from FCS results in a fluffy appearance of the CsCl. By contrast, sublimation of eutectic hexagonal ice/CsCl from FCS obtained after cooling produces a more spongy nature. These morphological differences also speak in favor of a previously unidentified phase appearing in CsCl solutions after vitrification by hyperquenching and after cold-crystallization by heating. By contrast, slower cooling methods do not feature this kind of fluffy morphology and also do not feature the path starting at very high-chemical potential but instead start from low chemical potential, barely allowing for the crystallization of metastable hydrates.

**Figure 10:**
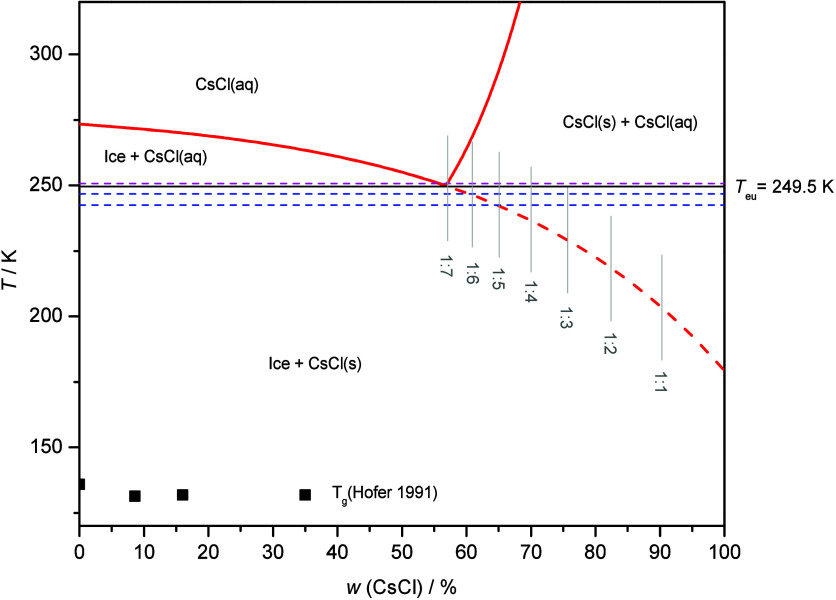
CsCl-H_2_O phase diagram. The freezing and solubility curves (solid red lines) were reconstructed from ref (8). The extended freezing curve (dashed red line) was constructed as an exponential decay fit of the freezing curve data. Blue dashed lines represent the onset temperatures of (presumed) CsCl hydrate eutectic melting as deduced from the DSC thermograph of the hyperquenched solution. We estimated the composition of the (presumed) hydrate at the intersection of the extrapolated freezing curve and the *T*_onset_ (HQ); it corresponds to a 65:35 weight ratio and 1:5 molar ratio of CsCl/H_2_O, respectively. The magenta dashed line represents the onset temperature of the eutectic melting of an ambient pressure LN-frozen sample (Figure 1). Here, the molar ratio of CsCl/H_2_O is 1:7.

This metastable nature and formation by nonequilibrium freezing conditions can be the reason for the discrepancy between our and Chen’s interpretation. For estimation of the composition of the metastable CsCl hydrate, we construct the extended CsCl-H_2_O phase diagram in Figure 10. The freezing and solubility curves (solid red lines) were adopted from ref (8). In order to describe the nonequilibrium freezing at high cooling rates, we have extended the freezing curve (dashed red line) as an exponential decay fit of the freezing curve data. The nonlinear curve fit was performed in Origin software using the ExpDec1 model, where parameters for the equation
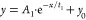
were sought by the least-squares method, giving the outcomes *A*_1_ = −5.84 ± 0.19, *t*_1_ = −35.21 ± 0.55, and *y*_0_ = 279.18 ± 0.21. One could attempt to estimate the composition of the potential hydrate from the intersection of the extended freezing line and the glass transition temperature *T*_g_ curves. However, *T*_g_ data for higher than 35 wt % CsCl are missing in the literature, making this approach not possible for the CsCl-hydrate case here. Instead, we assess the composition of the presumed hydrate from its eutectic melting temperature as deducted from the calorigram (Figure 1): a double peak starting at 242.5 and 246.7 K for both hyperquenched and pressurized hyperquenched samples and at 250.7 K for the frozen and pressure-amorphized sample. The onsets of the salt melting peaks in thermographs presented in Figure 1 are shifted toward a lower temperature for hyperquenched and pressurized-hyperquenched samples. These samples show previously unassigned Bragg peaks in XRD that are here considered to belong to the CsCl hydrates. Thus, we estimate the hydrate composition from the intersection of the extended freezing curve and the calorimetric onset melting temperature of the salt in the hyperquenched sample (*T*_onset_ (HQ) = 242.5 K). It corresponds to a 65:35 weight ratio, which gives the hydrate composition CsCl·5H_2_O. The other *T*_onset_ (246.7 K) in the thermograph of the hyperquenched sample (Figure 1) corresponds to the hydrate composition CsCl·6H_2_O (Figure 10). Thus, we assign the two types of metastable hydrate in the hyperquenched and pressurized hyperquenched samples to be the pentahydrate and hexahydrate, where the hexahydrate is the less metastable dominant hydrate and the pentahydrate, the most metastable hydrate that is the minor phase in the diffractograms and calorigrams

We identify unassigned Bragg peaks also in the XRD diffractograms of the ambient-pressure-cooled and pressure-amorphized samples (Figures 2 and 3), for which the calorigrams indicate melting temperatures of 249.5 K, very close to the eutectic temperature (Figure 1). The intersection of this temperature and the extended freezing curve in the phase diagram corresponds to a CsCl/water ratio of 1:7 (Figure 10). Thus, a hydrate of eutectic composition could be present in the nonhyperquenched samples. Regarding this, the results here are not fully consistent. While XRD shows metastable hydrate Bragg peaks after cooling in liquid nitrogen, the ESEM images also obtained after cooling in liquid nitrogen do not show the fluffy nature seen after sublimation of CsCl hydrates. In more slowly cooled samples, measured in the calorimeter metastable hydrate are also absent—which is expected based on the slow cooling that does not allow deviation from the thermodynamic expectations, meaning that the eutectic ice/CsCl mixture forms, but not the heptahydrate.

That is, in summary, we find evidence for the existence of the metastable pentahydrate and hexahydrate of CsCl. The metastable hydrates are feasible because of the ultrafast cooling employed here that leads to the fully glassy state and high chemical potential, very far from equilibrium. By contrast, slow cooling does not allow the formation of these hydrates because of near-equilibrium conditions all along the temperature path. Determination of the crystal structure of the metastable hydrates presented here remains a challenge—especially since they always form as a by-phase together with CsCl and hexagonal ice as main phases. It also remains unclear from the present work what the role of a network of veins buried within the ice is carrying the freeze-concentrated solution. However, we can clearly say that the FCS obtained upon cold-crystallization of the glass produces tiny, fine structures, while the FCS obtained upon simple cooling of the liquid produces larger, spongier structures. Particularly the former kind of FCS allows crystallizing CsCl-hydrates. We assume that the small amount of heptahydrate observed in the slow-cooling experiments forms in the veins containing FCS only, but we are unable to resolve that in the micrographs presently.

Our approach of making the metastable CsCl-hydrates has the potential of accessing many more molecules that are deemed unstable. The key to the success is turning the aqueous solution into the glass and then inducing cold-crystallization upon heating. The high-energy nature of the glass and the network of freeze-concentrated solution that emerges from it after cold-crystallization provide a chemical surrounding in which metastable molecules form well before the stable molecules known in chemistry textbooks. This is relevant in the laboratory for the synthesis of novel molecules. It is also of relevance in understanding how chemical reactions take place in space, where glassy aqueous solutions are abundant. Glassy aqueous solutions are the dominant way in which water is encountered in space, e.g., on interstellar dust grains or in comets. The cold-crystallization is triggered in such an environment upon the comet’s approach to the sun or in the process of the formation of a protoplanetary disc, which induces the formation of highly metastable molecular species and the evolution of molecules. That is, the freeze-concentrated solution observed here after cold-crystallization might actually be a birthplace for molecules in space.

## Data Availability

Data are available from the corresponding author T.L. upon reasonable request. Original ESEM images are available from V.N. upon reasonable request.
